# Calibration of camera internal parameters based on grey wolf optimization improved by levy flight and mutation

**DOI:** 10.1038/s41598-022-11622-w

**Published:** 2022-05-12

**Authors:** Daolei Wang, Jingwei Yue, Pingping Chai, Hao Sun, Feng Li

**Affiliations:** 1 College of Energy and Mechanical Engineering, Shanghai University of Electric Power, Shanghai, 200090 China; 2 School of Energy and Power Engineering, Xi’an Jiaotong University, Shaanxi, 710049 China

**Keywords:** Imaging and sensing, Computer science

## Abstract

Traditional calibration technology has been widely used in measurement and monitoring; however, there are limitations of poor calibration accuracy, which can not meet the accuracy requirements in some scenarios. About this problem, we proposed a grey wolf optimization algorithm based on levy flight and mutation mechanism to solve camera internal parameters in this paper. The algorithm is based on the actual nonlinear model, which takes the minimum average value of reprojection error as the objective function. The grey wolf position is randomly generated within a given range. Then, the grey wolf optimization algorithm based on levy flight and mutation mechanism is used to iteratively calculate the optimal position, which is the internal parameters of cameras. The two groups of experimental data were performed to verify the algorithm. The result shows better effectiveness and calibration accuracy of the proposed algorithm compared with other optimization methods.

## Introduction

With the improvement of artificial intelligence techniques, people's attention is focused on automatic driving, UAV patrol, robot navigation, 3D vision measurement, and other emerging fields^1–3^. Computer vision, including 3D measurement, is an essential component of artificial intelligence. Camera calibration, whose result will be involved in the next series of calculations, is a necessary process in vision measurement. As the first and most fundamental part of the calculation, it is essential for the accurate control of the entire system. For example, the calibration result of SLAM(simultaneous localization and mapping) directly affects the deviation between the reconstructed path and the actual path^4^. Therefore, camera calibration has become one of the most critical research areas in computer vision.

Camera calibration is a crucial steps of computer vision techniques in obtaining 3D information from 2D images^5^. The parameters of calibration are composed of internal parameters and external parameters. The external parameters are related to the motion state of the camera, which are variable quantities. In contrast the internal parameters are associated with the camera focal length, the distortion coefficient, and so on, which are physical attributes. Photos taken by the same camera have the same internal parameters. Therefore, the calibration is mainly concerned with internal parameters. According to the different nature of the established model, the methods can be sorted into the linear calibration method and nonlinear calibration method.

Linear calibration methods are based on linear imaging models, such as the direct linear transformation (DLT) method, which has the fewer calculation parameters; however, those methods do not take into account the nonlinear distortion caused by the lens and assembly process; therefore, the accuracy of the models has certain limitations^6^. Among the non-linear calibration methods, the calibration method of Zhang^7^ is widely used for simplicity and flexibility. This method is based on a nonlinear model and uses a two-dimensional checkerboard for calibration, and the maximum likelihood method is used to solve each parameter. The camera calibration problem is too complex, nonlinear, and multi-modal. Although the existing nonlinear methods have achieved good results, they often fall into local optimal solutions, and their accuracy is limited.

In current years, a few nature-inspired swarm intelligence optimization algorithms had been extensively carried out in visual calibration and detection, and performed the extraordinary effect and superiority, such as cuckoo algorithm (CS), whale algorithm (WOA), flower pollination algorithm (FPA), longicorn whisker algorithm (BAS), particle swarm optimization (PSO), dwarf mongoose optimization algorithm (DMO), ebola optimization search algorithmand (EOS), reptile search algorithm (RSA) so on. Some scholars gradually applied those methods to camera calibration, which can obtain the accuracy of camera parameters. Li, et al.^8^ proposed an effective camera calibrate method, which combined differential evolution way with PSO algorithm based on the camera calibration model of geometric parameters and lens distortion. Li, et al.^9^ proposed to combine the genetic algorithm and PSO algorithm to solve camera calibration problem, which can avoid particle swarm to the local optimal. Liu, et al.^10^ proposed an improved PSO algorithm with a new evaluation function, which adjusted the inertia factor and learning factor. This method achieved good results in camera internal parameter calibration. Qin et al.^11^ proposed a full-parameter PSO algorithm based on mutation strategy, improving convergence speed and accuracy compared with the traditional particle swarm calibration ways. Yang, et al.^12^ utilized the simulated annealing algorithm and the PSO algorithm to combine the advantages of those methods through a collaborative mechanism, which the hybrid algorithm can merge the advantages of different methods. the method produces more good performance than a single algorithm, which can improve the accuracy of the calibration results.

The preceding observation^13^ suggests that the results of calibration is affected by the accuracy of feature extraction. Thus, numerous researchers have concentered on creating targets with distinctive features that can be accurately localized in the images, including 1D, 2D and 3D targets. Among those targets, 3D targets are difficultly created and hardly solved. Therefore, it limits their applications. The advantage of 1D targets is flexibility, however, due to the fewer feature point involved, they are hard to acquire high accuracy. In contrast, the second objectives have been broadly investigated attributed to their flexibility and effectiveness. There are two typically used patterns of 2D targets: checkerboards and circles. However, in some scenarios, the calibration accuracy of 2D targets is still limited. In this paper, an improved grey wolf algorithm based on Levy flight and mutation strategy is designed to solve camera calibration; and then the objective model is optimized by this method to acquire the accuracy parameters of camera. The error of calibration parameter’s results obtained is smaller than the grey wolf algorithm, particle swarm optimization algorithm, and Zhang’s calibration method^7^.

## Monocular camera model and four coordinate systems

**Figure 1:**
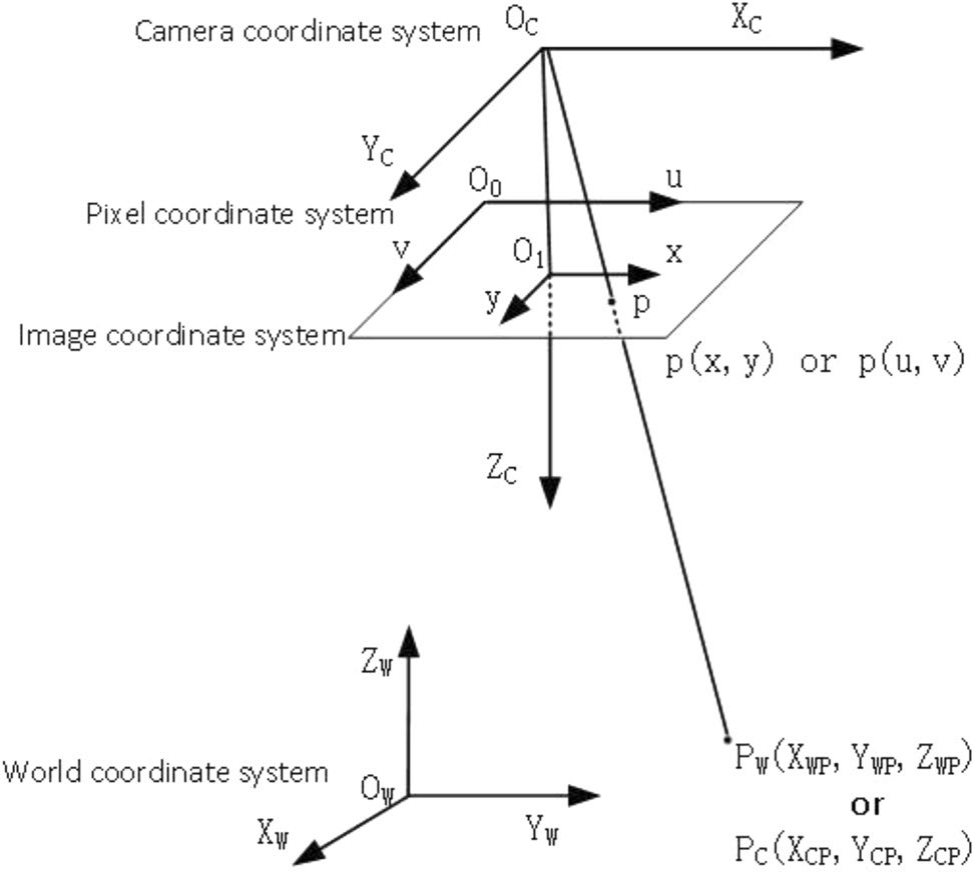
Monocular camera model.

Camera imaging model, which mainly reflects the process of the camera taking pictures of the actual 3D world. The model of camera imaging is the foundation of camera calibration and the solution camera parameters. According to the difference of model of camera imaging, it can be categorized into linear and nonlinear model. The linear model is based on the pinhole imaging precept, which establishes the relationship of pinhole imaging point and the corresponding object point through its geometric courting^14^. The camera distortions are usually caused by the physical structure in practical applications. Therefore, a nonlinear model may be applied to correct the camera distortion. The cameral imaging model is shown in Fig. 1.

Imaging includes four coordinate systems. They are called World Coordinate System (WCS), Camera Coordinate System (CCS), Image Coordinate System (ICS) and Pixel Coordinate System (PCS). They are converted according to a certain scale.

World coordinate system $\left( {X_{W} ,Y_{W} ,Z_{W} } \right)$, which is also called measurement coordinate system. It is an orthogonal 3D rectangular coordinate system, which is established based on an object in reality.$\left( {X_{WP} ,Y_{WP} ,Z_{WP} } \right)$ denotes the coordinate of object point $P$ in the world coordinate system.

Camera coordinate system $\left( {X_{C} ,Y_{C} ,Z_{C} } \right)$: It is a 3D rectangular coordinate system. The origin is located in the optical center of the camera. The axis $Z_{C}$ is the main optical axis of the camera.$\left( {X_{CP} ,Y_{CP} ,Z_{CP} } \right)$ is the image coordinate of object point $P$ in the ideal pinhole model.

Image coordinate system $\left( {x,y} \right)$: A 2D rectangular coordinate system established on the image plane. The transformation between the CCS and ICS is known as perspective transformation. The coordinate axis $x$ and $y$ are respectively parallel to the axis $X_{{\text{c}}}$ and $Y_{c}$.The origin $O_{1}$ is situated in the intersection of the camera optical axis and the CCD image plane. $\left( {x_{P} ,y_{P} } \right)$ denotes the coordinate of object point $P$ in the image coordinate system.

Pixel coordinate system $\left( {u,v} \right)$:It is established on the same plane as the image coordinate system, while the difference is that the origin of the two is different. The coordinate axis $x$ and $y$ are respectively parallel to the axis u and v. $\left( {u_{P,} v_{P} } \right)$ denotes the pixel coordinate of object point $P$ in the pixel coordinate system.

### External parameters of the camera

Rigid body transformation describes the relative positional relationship between the world coordinate system (WCS) and the camera coordinate system (CCS) through the external parameters of the camera. Assume that there is a point $P$ in three-dimensional space in reality, and its coordinate in the world coordinate system is $\left( {X_{WP} ,Y_{WP} ,Z_{WP} } \right)$. The formula (1) shows the coordinates $\left( {X_{CP} ,Y_{CP} ,Z_{CP} } \right)$ of point $P$ when it's converted to camera coordinates.

1 $$\left[ {\begin{array}{*{20}c} {X_{CP} } \\ {Y_{CP} } \\ {Z_{CP} } \\ 1 \\ \end{array} } \right] = \left[ {\begin{array}{*{20}c} {{\varvec{R}}_{3*3} } & {{\varvec{T}}_{3*1} } \\ 1 & 0 \\ \end{array} } \right]\left[ {\begin{array}{*{20}c} {X_{WP} } \\ {Y_{WP} } \\ {Z_{WP} } \\ 1 \\ \end{array} } \right]$$

where ${\varvec{R}}$ and ${\varvec{T}}$ respectively represent the rotation matrix and translation vector. And they do not depend on the inherent attributes of the camera itself. It shows there are two external parameters involved in camera calibration.

### Internal parameters of the camera

If the error caused by distortion is not taken into account, as shown in Fig. 1, camera model is a linear model. And then the geometric knowledge can be used to solve the relationship between parameters. The point coordinates of camera $\left( {X_{CP} ,Y_{CP} ,Z_{CP} } \right)$ is calculated by external parameters. After a perspective transformation, the points on the physical coordinate system of the image are obtained as $\left( {x_{P} ,y_{P} } \right)$.

2 $$\left[ {\begin{array}{*{20}c} {x_{P} } \\ {y_{p} } \\ 1 \\ \end{array} } \right] = \left[ {\begin{array}{*{20}c} {f/Z_{CP} } & 0 & 0 & 0 \\ 0 & {f/Z_{CP} } & 0 & 0 \\ 0 & 0 & 1 & 0 \\ \end{array} } \right]\left[ {\begin{array}{*{20}c} {X_{CP} } \\ {Y_{CP} } \\ {Z_{CP} } \\ 1 \\ \end{array} } \right]$$

where $f$ is the camera’s focal length.

Imaging in the image pixel coordinate system is the last presentation form of the camera. Pixel coordinates $\left( {u,v} \right)$ can be obtained directly from a photograph. Also taking the point $P$ as an example. The transformation between the pixel point and the image physical coordinate system in the same imaging plane with a different origin can be described by the following formula:

3 $$\left[ {\begin{array}{*{20}c} {u_{P} } \\ {v_{P} } \\ 1 \\ \end{array} } \right] = \left[ {\begin{array}{*{20}c} {1/d_{x} } & 0 & {u_{0} } \\ 0 & {1/d_{y} } & {v_{0} } \\ 0 & 0 & 1 \\ \end{array} } \right]\left[ {\begin{array}{*{20}c} {x_{P} } \\ {y_{P} } \\ 1 \\ \end{array} } \right]$$

where $\left( {u_{0} ,v_{0} } \right)$ is the intersection point of the two coordinate axes, namely the coordinates of the origin $O_{0}$, $d_{x}$ and $d_{y}$ represents the physical size of the unit pixel on the two coordinate axes.

In combination with (1), (2) and (3), it can be obtained that:

4 $$\left\{ {\begin{array}{*{20}l} {K = \left[ {\begin{array}{*{20}c} {1/d_{x} } & 0 & {u_{0} } \\ 0 & {1/d_{y} } & {v_{0} } \\ 0 & 0 & 1 \\ \end{array} } \right]\left[ {\begin{array}{*{20}c} {f/Z_{CP} } & 0 & 0 & 0 \\ 0 & {f/Z_{CP} } & 0 & 0 \\ 0 & 0 & 1 & 0 \\ \end{array} } \right] = \left[ {\begin{array}{*{20}c} {f_{x} } & \gamma & {u_{0} } \\ 0 & {f_{y} } & {v_{0} } \\ 0 & 0 & 1 \\ \end{array} } \right]} \hfill \\ {F = \left[ {\begin{array}{*{20}c} {{\varvec{R}}_{3*3} } & {{\varvec{T}}_{3*1} } \\ 1 & 0 \\ \end{array} } \right]} \hfill \\ {z_{c} \left[ \begin{gathered} u_{P} \hfill \\ v_{P} \hfill \\ 1 \hfill \\ \end{gathered} \right] = KF\left[ {\begin{array}{*{20}c} {X_{WP} } \\ {Y_{WP} } \\ {Z_{WP} } \\ 1 \\ \end{array} } \right]} \hfill \\ \end{array} } \right.$$

where $K$ and $F$ are the internal parameter matrix and the external parameter matrix respectively.

### Solving camera parameters

So as to simplify the calculation, it’s miles assumed that the template plane is on $Z_{w}$ = 0 in WCS.

Formula (4) can be simplified as below:

5 $$z_{c} \left[ \begin{gathered} u_{P} \hfill \\ v_{P} \hfill \\ 1 \hfill \\ \end{gathered} \right] = K\left[ {r1 \, r2 \, t} \right]\left[ \begin{gathered} X_{WP} \hfill \\ Y_{WP} \hfill \\ 1 \hfill \\ \end{gathered} \right]$$

In order to solve camera internal parameters, a new concept called homography matrix is introduced.

Truly understood as it is used to explain the position mapping relationship between the object within the world coordinate system and the pixel coordinate system. The corresponding transformation matrix is the homography matrix, which is expressed as:

6 $$H = sK\left[ {r_{1} \, r_{2} \, t} \right]$$

where $K$ is same as formula (4), which is the internal parameter matrix.$s$ is the scale factor.

If assume $H^{ - 1} = \left[ {h_{11} ,h_{12} ,h_{13} ;h_{21} ,h_{22} ,h_{23} ;h_{31} ,h_{32} ,h_{33} } \right]$ ,so $\left( {X_{WP} ,Y_{WP} } \right)$ can be calculated below:

7 $$\left\{ {\begin{array}{*{20}l} {X_{WP} = \frac{{u_{P} h_{11} + v_{P} h_{12} + h_{13} }}{{u_{P} h_{31} + v_{P} h_{32} + h_{33} }}} \hfill \\ {Y_{WP} = \frac{{u_{P} h_{21} + v_{P} h_{22} + h_{23} }}{{u_{P} h_{31} + v_{P} h_{32} + h_{33} }}} \hfill \\ \end{array} } \right.$$

Then, the expression can be expressed as:

8 $$\left[ \begin{gathered} X_{WP} \, Y_{WP} \, 1 \, 0 \, 0 \, 0 \, - u_{P} X_{WP} \, - u_{P} Y_{WP} \, - u_{P} \hfill \\ 0 \, 0 \, 0 \, X_{WP} \, Y_{WP} \, 1 \, - v_{P} X_{WP} \, - v_{P} Y_{WP} \, - v_{P} \hfill \\ \end{gathered} \right]h = 0$$

Because the image point in the central area of the image is less affected by distortion factors. Therefore, in order to reduce the calculation error, select the image center area $n$ (*n* > 9) to pixel points $\left( {u_{P} ,v_{P} } \right)$ and $\left( {X_{WP} ,Y_{WP} ,0} \right)$, the matrix $H$ can be calculated. According to the definition of $H$ in formula (6), $r1$ and $r{2}$ are orthogonal vectors, thus:

9 $$\left\{ \begin{gathered} r1^{T} r1 = r2^{T} r2 = 1 \hfill \\ r1^{T} r2 = 0 \hfill \\ \left\| {r1} \right\| = \left\| {r2} \right\| = 0 \hfill \\ \end{gathered} \right.$$

Namely:

10 $$\left\{ \begin{gathered} H_{1}^{T} BH_{2} = 0 \hfill \\ H_{1}^{T} BH_{1} = H_{2}^{T} BH_{2} \hfill \\ \end{gathered} \right.$$

where $B = K^{ - T} K^{ - 1}$,and is a symmetric matrix, which can be defined as a 6-dimensional vector $b = \left[ {B_{11} \, B_{12} \, B_{22} \, B_{13} \, B_{23} \, B_{33} } \right]^{T}$. From Eq. (10), The camera intrinsic parameters are determined by the two main constraints, which can be expressed as follows:

11 $$\left[ \begin{gathered} N_{12}^{T} \hfill \\ \left( {N_{11} - N_{22} } \right)T \hfill \\ \end{gathered} \right]b = 0$$

where $$N_{ij} = \left[ \begin{gathered} h_{i1} h_{j1} \\ h_{i1} h_{j1} + h_{i2} h_{j1} \\ h_{i2} h_{j2} \\ h_{i3} h_{j1} + h_{i1} h_{j3} \\ h_{i3} h_{j2}^{{}} + h_{i2} h_{j3} \\ h_{i3} h_{j3} \\ \end{gathered} \right]$$. In Eq. (11), $N_{ij}$ is known; The matrix $B$ contains 6 unknowns and requires 6 equations to solve. One homography matrix $H$ can express two equations, for which three calibration pictures with different shooting angles are needed to solve $B$. Then, matrix $B$ is decomposed by Cholesky to obtain the camera's internal parameter matrix $K$. The specific solution method can be referred to paper^8^. According to the homography $H$ and intrinsic parameters, camera extrinsic parameter can be solved as follows:

12 $$\left\{ \begin{gathered} r1 = sK^{ - 1} h_{1} \hfill \\ r2 = sK^{ - 1} h_{2} \hfill \\ r3 = r1 \times r2 \hfill \\ t = sK^{ - 1} h_{3} \hfill \\ s = \frac{1}{{\left\| {K^{ - 1} h_{1} } \right\|}} = \frac{1}{{\left\| {K^{ - 1} h_{2} } \right\|}} \hfill \\ \end{gathered} \right.$$

where the scale factor $s$ is determined by the orthogonal condition. So far, the initial internal and external parameters of the camera have all been solved.

### Distortion correction

Note that lens distortion of the camera became no longer considered throughout the above calibration system. The distortion resulting from the lens shape is referred to as radial distortion. Within the pinhole model, a straight line projected onto the pixel plane remains a straight line. however, in actual photos, the digicam lens regularly turns a straight line within the actual environment into a curve in the picture. The closer to the edge of the picture, the more obvious this phenomenon. For the reason that actual processed lens is often symmetrical, irregular distortion is generally radially symmetrical. They specifically divided into two categories, barrel distortion and pincushion distortion.

Barrel distortion is because the photo magnification decreases as the distance from the optical axis will increase, while pincushion distortion is simply the opposite. In those sorts of distortions, the instantly line passing through the intersection of the picture center and the optical axis can keep its form. Similarly, to the radial distortion brought via the form of the lens, tangential distortion can also be delivered due to the inability to make the lens and the imaging surface strictly parallel at some point of the meeting system of the digicam^15^.

In summary, distortion is mainly classified into three types: eccentric, radial and thin prism distortion. Under the comprehensive action, there will be errors in both radial and tangential directions, and the error calculation formula is as follows:

13 $$\left\{ \begin{gathered} x^{2} + y^{2} = r^{2} \hfill \\ \partial_{y} \left( {x,y} \right) = p_{1} r^{2} + y\left( {2p_{1} y + 2p_{2} x + k_{1} r^{2} + k_{2} r^{4} + k_{3} r^{6} + 1} \right) \hfill \\ \partial_{x} \left( {x,y} \right) = p_{2} r^{2} + x\left( {2p_{1} y + 2p_{2} x + k_{1} r^{2} + k_{2} r^{4} + k_{3} r^{6} + 1} \right) \hfill \\ \end{gathered} \right.$$

where $k_{1} ,k_{2} ,p_{1} ,p_{2} ,k_{3}$ are the five variables, which are the factors related to the distortion correction in both directions.

Here the nonlinear model introduced distortion is used to calibrate the camera.

In summary, the distortion factor $\left( {k_{1} ,k_{2} ,p_{1} ,p_{2} ,k_{3} } \right)$, the parameters related to the focal length $\left( {f_{x} ,f_{y} } \right)$, and the image center $\left( {u_{0} ,v_{0} } \right)$ are the nine internal parameters of the camera in total.

## Improved grey wolf optimization algorithm

### Wolf optimization algorithm

**Figure 2:**
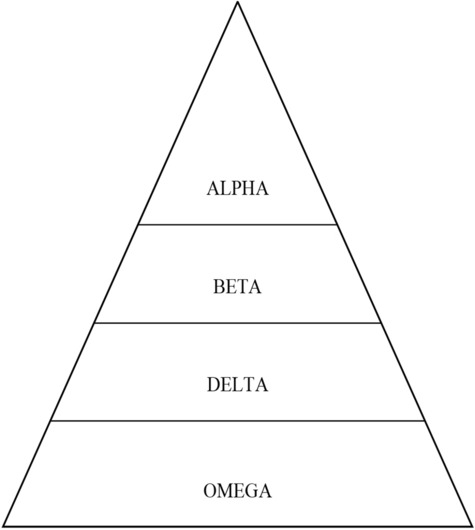
Social hierarchy of wolves and their characteristics in GWO.

Grey Wolf Optimization Algorithm (GWO) is a recent population-based bionic algorithm, which is given by Mirjalili^16^. This algorithm simulates social hunting conduct of grey wolves. In comparison with different meta-heuristic algorithms, the GWO algorithm can be definitely understood as an efficient optimization technique that simulates the social behavior and management hierarchy of grey wolves. In the mathematical instance of the hierarchy of the grey wolves, alpha(α) wolf is referred to as the most suitable solution. consequently, beta(β) wolf is referred to as the second one most appropriate solution after which the following appropriate solution is known as delta(δ). the alternative populations which constitute the furthest solutions are seemed to be the omegas (ω)^17^. The quality wolves must be dealt with as α, β, and δ that help exceptional wolves (ω) in exploring more favorable regions of solution area, as shown in Fig. 2.

The grey wolves encircle the victim while hunting. The surrounding strategy, is mathematically represented as in(14):

14 $$\left\{ \begin{gathered} \overrightarrow {X} \left( {k + 1} \right) = \overrightarrow {{X_{p} }} \left( k \right) - \overrightarrow {A} * \vec{B} \hfill \\ \overrightarrow {A} = 2a\overrightarrow {r}_{1} - a \hfill \\ \overrightarrow {B} = \left| {\overrightarrow {D} * \overrightarrow {{X_{p} }} \left( k \right) - \overrightarrow {X} \left( k \right)} \right| \hfill \\ \overrightarrow {D} = 2\overrightarrow {{r_{2} }} \hfill \\ \end{gathered} \right.$$

where $\overrightarrow {A}$ and $\overrightarrow {D}$ represent coefficient vectors. $\overrightarrow {X} \left( k \right)$ and $\overrightarrow {X}_{p} \left( k \right)$ represent location of wolves and location of the prey respectively.$k$ shows recent iteration.$a = 2 - \frac{k}{{k_{\max } }}$ is a temporal parameter that its elements are linearly decreased from 2 to 0. $\vec{r}_{1}$ and $\vec{r}_{2}$ are random vectors between [0, 1].

These animals need to discover and enclose the location of the quarry. The searching technique is usually directed through the alpha types. In a few conditions, the beta and delta would possibly contribute in searching as well. In GWO, it has been intended that the alpha (best solution), beta and delta can guesstimate the in all likelihood scenario of the sufferer. Hence, the primary three best solutions should be recorded to conduct other hunters. It's miles clear that the end position is located in a random vicinity inside a hypersphere within the search space. Therefore, elite solutions will approximate the top of the line and other hunt retailers simply ought to revise their locations around a predicted victim based totally on stochastically.

After encircling the prey, the wolves hunt the prey under the leadership of the alphas, betas and deltas^18^. The position of the prey also changes as it runs away. At this time, the positions of the wolves change accordingly, and the calculation formula is as follows:

15 $$\left\{ \begin{gathered} \overrightarrow {{X_{1} }} \left( {k + 1} \right) = \overrightarrow {{X_{\alpha } }} \left( k \right) - \overrightarrow {{A_{1} }} * \overrightarrow {{B_{\alpha } }} \hfill \\ \overrightarrow {{B_{\alpha } }} = \left| {\overrightarrow {{D_{1} }} * \overrightarrow {{X_{\alpha } }} \left( k \right) - \overrightarrow {X} \left( k \right)} \right| \hfill \\ \end{gathered} \right.$$

16 $$\left\{ \begin{gathered} \overrightarrow {{X_{2} }} \left( {k + 1} \right) = \overrightarrow {{X_{\beta } }} \left( k \right) - \overrightarrow {{A_{2} }} * \overrightarrow {{B_{\beta } }} \hfill \\ \overrightarrow {{B_{\beta } }} = \left| {\overrightarrow {{D_{2} }} * \overrightarrow {{X_{\beta } }} \left( k \right) - \overrightarrow {X} \left( k \right)} \right| \hfill \\ \end{gathered} \right.$$

17 $$\left\{ \begin{gathered} \overrightarrow {{X_{3} }} \left( {k + 1} \right) = \overrightarrow {{X_{\delta } }} \left( k \right) - \overrightarrow {{A_{3} }} * \overrightarrow {{B_{\delta } }} \hfill \\ \overrightarrow {{B_{\delta } }} = \left| {\overrightarrow {{D_{3} }} * \overrightarrow {{X_{\delta } }} \left( k \right) - \overrightarrow {X} \left( k \right)} \right| \hfill \\ \end{gathered} \right.$$

18 $$\overrightarrow {X} \left( {k + 1} \right) = \frac{{\overrightarrow {{X_{1} }} + \overrightarrow {{X_{2} }} + \overrightarrow {{X_{3} }} }}{3}$$

where $\overrightarrow {{B_{\alpha } }}$, $\overrightarrow {{B_{\beta } }}$, $\overrightarrow {{B_{\delta } }}$ represent the distance vector between the alpha wolf, beta wolf, delta wolf and other wolves respectively;$\overrightarrow {{X_{1} }}$, $\overrightarrow {{X_{2} }}$, $\overrightarrow {{X_{3} }}$ represent updated position vectors under the leadership of the alpha wolf, beta wolf, delta wolf ; $\overrightarrow {X} (k + 1)$ is the final position vector.

### Levy flight

Levy flight is a type of walk that follows Levy probability distribution function and arbitrarily orientations the step length^19^. In addition, Levy flight has been found to be widespread in nature. Creatures such as sharks and cuckoos move according to this law. This distribution is a simple power -law formula. The formula is usually as follows:

19 $$L\left( {s,\gamma ,\mu } \right) = \left\{ {\begin{array}{*{20}l} {\sqrt {\frac{\gamma }{2\pi }} \exp \left[ { - \frac{\gamma }{{2\left( {s - \mu } \right)}}} \right]\frac{1}{{\left( {s - \mu } \right)^{\frac{3}{2}} }}} \hfill & {if\;0 < \mu < \infty } \hfill \\ 0 \hfill & {otherwise} \hfill \\ \end{array} } \right.$$

where $s$ is step length, $\mu$ is parameter is location or shift parameter,,$\gamma$, $L\left( s \right)$ represents the distribution of $s$.$\gamma > 0$ parameter is scale (controls the scale of distribution) parameter.

Levy distribution can be represented by a clear power-law equation as:

$$L\left( s \right) \sim \left| s \right|^{ - 1 - \beta } 0 < \beta \le 2$$

In the Mantegna strategy, the normal distribution is used to solve the random step size, and the method is as follows:

20 $$s = \frac{u}{{\left| \nu \right|^{{\frac{1}{\beta }}} }}$$

where $u$ and $v$ are drawn from normal distributions.

**Figure 3:**
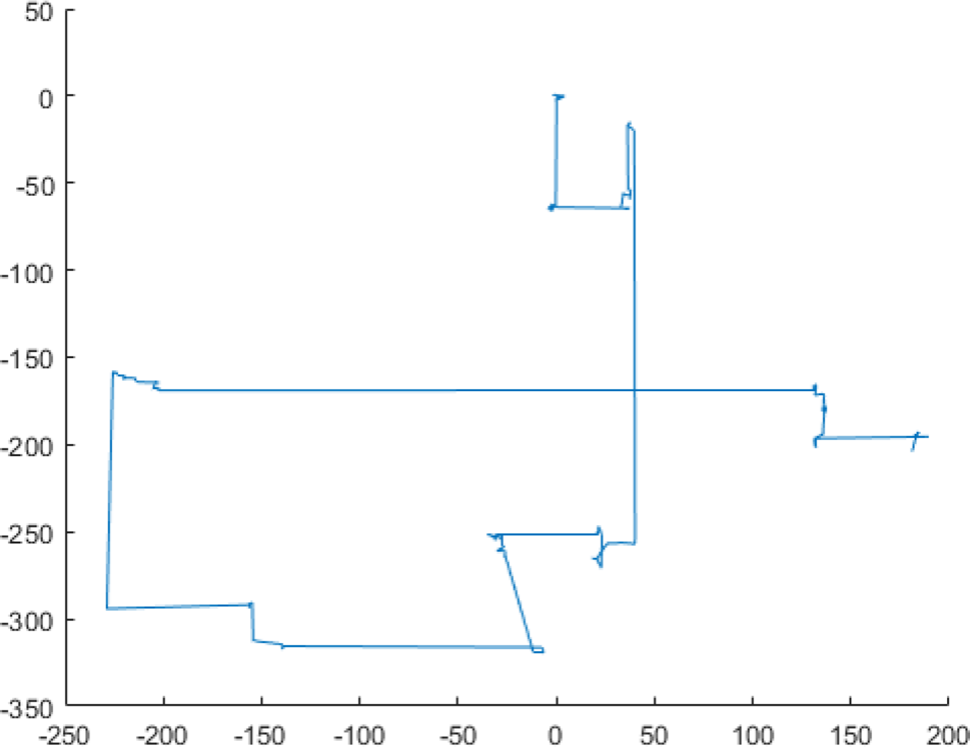
Levy flight simulation trajectory.

The result of levy flight simulation is shown in Fig. 3. It can be obviously seen from the figure that this random walk can produce high-frequency short step lengths and intermittent long step lengths. This feature can suppress the shortcomings of the grey wolf algorithm that is not difficult to fall into local extremes, and will not weaken the ability of global optimization.

### Grey wolf optimization improved by Levy flight and mutation mechanism

When using swarm intelligence algorithms to solve complex high-dimensional global optimization problems, balancing algorithm convergence and population diversity is very important. As a swarm intelligence algorithm, GWO also is required to solve this problem. Mutation can promote the evolution of living organisms and bring next-generation individuals more adaptable to the environment. The bionic structure of the GWO algorithm gives it the advantages of realization and flexibility. However, the diversity of individuals in the later stage of GWO is insufficient, and the range of prey hunting will be stagnant in the local Optimal area^20^. To maintain the diversity of the following individuals and jump out of the local optimal area, this paper generates grey wolf intermediates according to the mutation mechanism in the differential evolution algorithm, as showed in Eq. (21):

21 $${\text{G}}_{i} \left( {k + 1} \right) = X_{\alpha } \left( k \right) + F\left( {X_{r1} \left( k \right) - X_{r2} \left( k \right)} \right)$$

where $G_{i} (k + 1)$ is intermediate,$i \ne r_{1} \ne r_{2}$, $X_{\alpha }$ is the optimal solution, $X_{r2}$ and $X_{r3}$ are 2 position vectors randomly selected in a population.$F$ is the scaling factor, 0.5 here.

In the grey wolf optimization algorithm, the state of the grey wolf can be judged by $\left| {\vec{A}} \right|$: when $\left| {\vec{A}} \right| > 1$, the wolves are far away from the prey, global search is conducted; when $\left| {\vec{A}} \right| < 1$, the wolves are close to the prey, local search is conducted. Therefore, this paper is based on the formula (13), in order to balance the local and Global optimization capability, combined with Levy flight, the formula is as follows:

22 $${\text{G}}_{i} \left( {k + 1} \right){ = }\left\{ {\begin{array}{*{20}l} {X_{\alpha } \left( k \right) + F\left( {X_{r1} \left( k \right) - X_{r2} \left( k \right)} \right),} \hfill & {\left| {\overrightarrow {A} } \right| < 1} \hfill \\ {X_{\alpha } \left( k \right) + F\left( {X_{r1} \left( k \right) - X_{r2} \left( k \right)} \right) + \varepsilon \oplus Levy\left( s \right),} \hfill & {\left| {\overrightarrow {A} } \right| > 1} \hfill \\ \end{array} } \right.$$

where $\varepsilon$ is the step control factor, here is 0.01.

The intermediate is obtained through the above formula and then compared with the original individual, and the optimal position is updated according to the greedy (GS) algorithm.

23 $$X_{i} \left( {k + 1} \right) = \left\{ {\begin{array}{*{20}l} {{\text{G}}_{i} \left( {k + 1} \right),} \hfill & {f\left( {X_{i} \left( k \right)} \right) > f\left( {{\text{G}}_{i} \left( {k + 1} \right)} \right)} \hfill \\ {X_{i} \left( k \right),} \hfill & {f\left( {X_{i} \left( k \right)} \right) < f\left( {{\text{G}}_{i} \left( {k + 1} \right)} \right)} \hfill \\ \end{array} } \right.$$

The optimal individual is chosen at each iteration to achieve optimization purpose.

**Figure 4:**
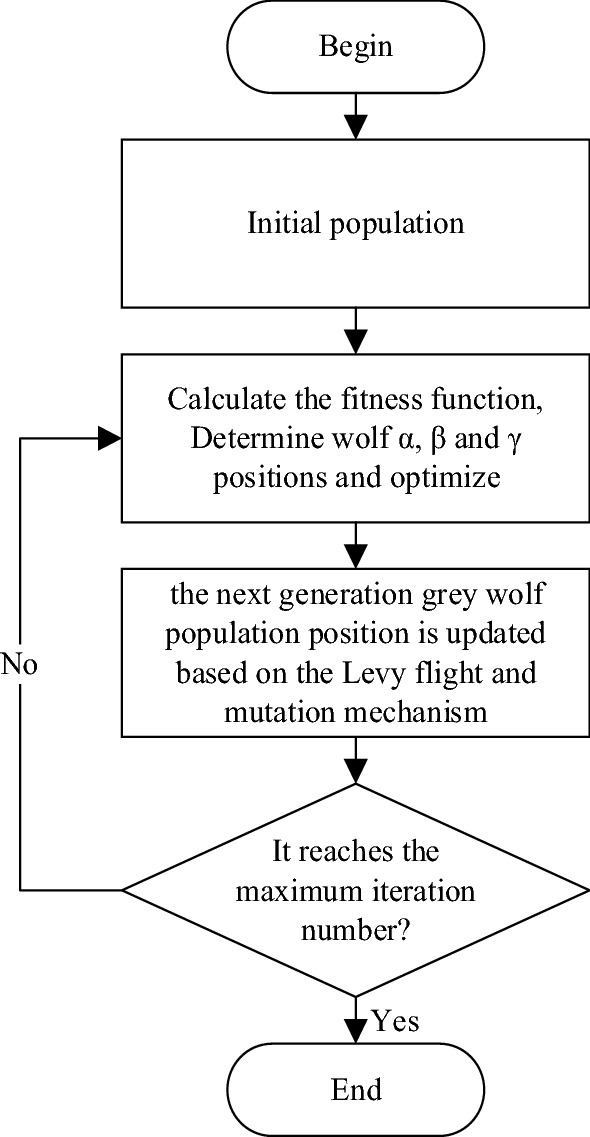
The flow chart of the improved grey wolf algorithm.

In summary, the algorithm is summarized as follows, and the first step is to initialize the position. The flowchart is shown in Fig. 4.

The detailed pseudo code of the improved GWO algorithm is shown in algorithm 1.
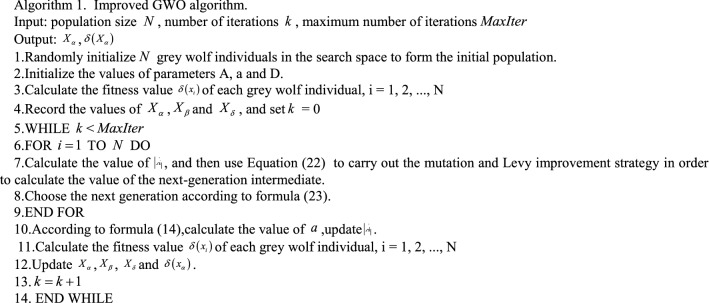


Compared to the basic GWO algorithm, the improved grey wolf algorithm has the following characteristics: (1) Improved grey wolf algorithm introduces levy flight to balance global exploration and local mining capabilities; (2)Implement the mutation strategy for the current individual to enhance the diversity of the population and reduce the probability of the algorithm falling into local optimum.

## Experimental results and analysis

The experimental platform is a notebook with 8G memory and Core i5. The program is implemented in PYTHON language and tested on public and self-made photo collections.

Through the evaluation of the camera model, the proposed improved grey wolf algorithm is implemented to obtain camera parameters. The 2-dimensional coordinates of the acquired image are as compared with the actual measured image coordinates to confirm the feasibility and superiority of the algorithm.

- *Step 1*.To get the actual pixel coordinates in the image by Using PYTHON-OPENCV.
- *Step 2*. Based on the parameters obtained by Zhang’s method^7^, determine the upper and lower bounds of the iteration. In order to avoid slow convergence caused by too large optimization range, the obtained internal parameters ± 80 and distortion coefficient ± 10 are used as the upper and lower bounds of the optimization. Set the number of grey wolves in the wolf algorithm is 40, and it runs 400 times in total. The objective function is established, that is, the average value of the pixel distance between the actual coordinates and the optimized reprojection. Assuming we have N calibration points. Each point is identical in size and placed in the same noise environment. Objective function is established as follows:
  $$\delta = \frac{1}{N}\sum\limits_{{j = 1}}^{N} {\left\| {p_{j} - p_{j}^{\prime } \left( {k_{1} ,k_{2} ,p_{1} ,p_{2} ,k_{3} ,R,T,f_{x} ,f_{y} ,u_{0} ,v_{0} } \right)} \right\|^{2} }$$
  where $N$ Denotes the number of calibrated corner points,$p_{j}$ represents the actual pixel coordinates of corner $j$, and $p^{^{\prime}}_{j}$ represents the reprojection coordinates of corner $j$. $R$ and $T$ are the rotation matric and translation vector corresponding to the *j*th image.
- *Step 3*. Randomly generate an initial value between the upper and lower bounds, and the initial value is 9 dimensions.
- *Step 4*. Calculate the value of $f$, obtain the wolves positions of $\alpha$, $\beta$ and $\gamma$ according to the sorting result, and then optimize.
- *Step 5*. Update the final position of the movement according to Eq. (14) and (15).
- *Step 6*. If the iteration number reaches the maximum number, the iteration will be stopped; else return to step 4 to continue running.
- *Step 7*. Finally, the best individual position and the best function value are returned, and the output is the mean value of the calibration error.

### Experiments on public data sets

According to the article^11^, experiment in the public picture collection. The series of photos are from the sample pictures of the calibration toolbox (http://www.vision.caltech.edu/bouguetj/calib_doc/htmls/example.html). The size of the grid is 30mmx30mm. Each picture has 182 corner points. The unequal row–column dimension is adopted, which can always determine the direction of chessboard corner detection and ensure the corner correct management. The internal calibration results are shown in the Table 1 below:

**Table 1:** Calculation results of the method in this paper.

Parameters	Result
$f_{x}$	664.832 5
$f_{y}$	668.946 6
$u_{0}$	300.000 0
$v_{0}$	236.122 2
$k_{1}$	− 0.300 1
$k_{2}$	0.199 7
$p_{1}$	− 0.000 8
$p_{2}$	0.002 2
$k_{3}$	− 1.300 1
$\delta$/pixel	0.102

As can be seen from the Table 1, the average error value of the proposed method in this paper is 0.102 pixel, which is better than the 0.17 pixel in the paper^12^ and improves the calibration accuracy to a certain extent.

**Figure 5:**
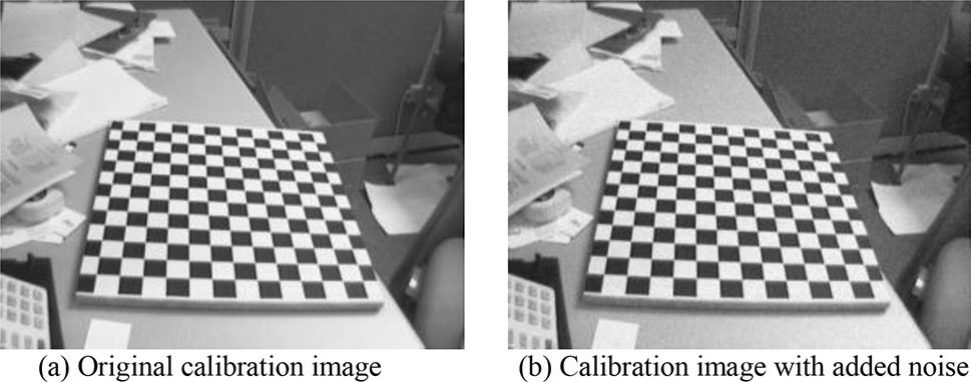
Comparison of the original calibration image and the image with added noise.

**Figure 6:**
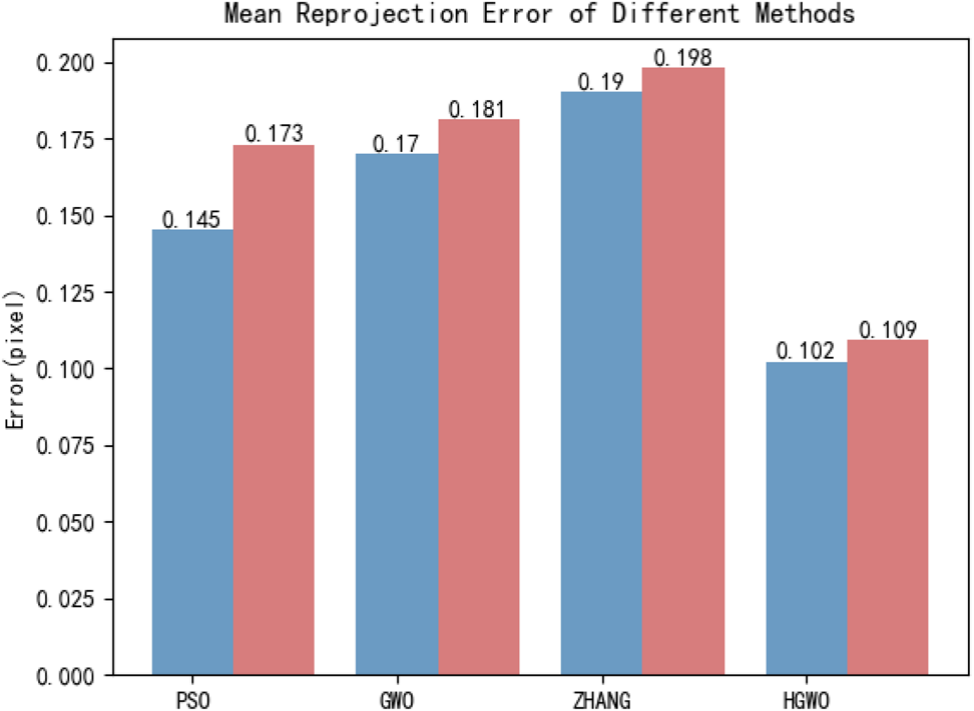
Comparison of the original calibration image and the image with added noise.

In order to test the robustness of the algorithm, noise was added to the sample images and then compared with the calibration results of grey Wolf algorithm, particle swarm optimization algorithm and Zhang’s calibration method. Examples and results of images with noise are shown in Figs. 5 and 6 respectively.

As can be observed in from Fig. 6, compared with the original image calibration results at the beginning, the average calibration error of the method in this paper has been increased by 0.07 pixels after adding noise, but the error is still the lowest in each method.

Two experiments show that the proposal algorithm improves the calibration accuracy effectively and has the characteristics of stability.

### Experiments on self-made data sets

**Figure 7:**
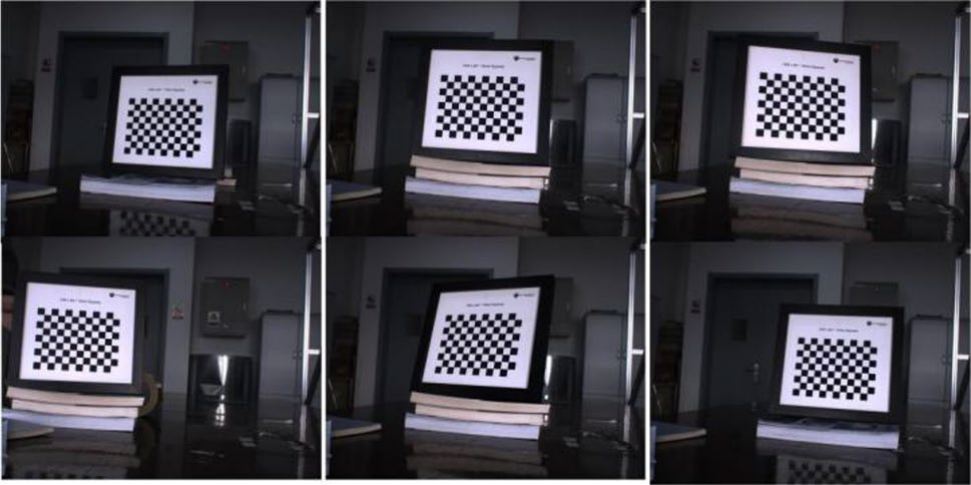
Pictures collected by the camera.

In the experiment, the AVT Manta G-201B camera produced by AVT in Germany was used to capture a new set of photos of a laser-printed two-dimensional checkerboard. The size of the checkerboard is 11 × 8, and the grid size is 15mmx15mm. A photo can capture 88 corners. A total of 15 photos were collected, and the collected photos are shown in Fig. 7.

**Figure 8:**
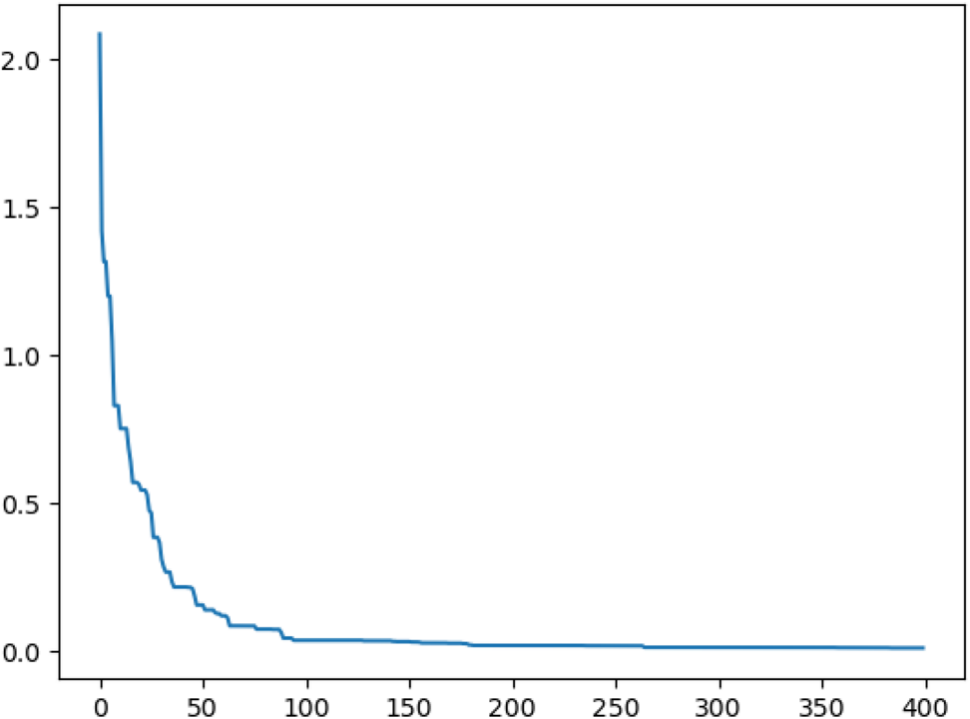
The objective function curve obtained by the improved grey wolf algorithm.

Figure 8 shows the change of the objective function based on the hybrid algorithm in the process of 400 iteration. Table 2 lists the different results obtained by the four methods.

**Table 2:** Calculation results of different algorithms.

Parameters	Ours	Zhang	PSO	GWO
$f_{x}$	1890.414	1894.506	1886.405	1894.540
$f_{y}$	1891.191	1895.103	1887.624	1895.147
$u_{0}$	803.550	799.712	803.761	803.342
$v_{0}$	629.425	634.544	629.419	629.615
$k_{1}$	− 0.057 6	− 0.1012	0.049 9	− 0.090 8
$k_{2}$	0.000 2	0.194 2	− 1.000 0	0.171 3
$p_{1}$	− 0.000 6	/	0.000 1	− 0.004 9
$p_{2}$	0.000 1	/	− 0.000 8	0.002 8
$k_{3}$	− 0.099 8	/	0.001 2	0.074 5
$\delta$/pixel	0.026	0.105	0.058	0.075

As can be seen from the figure above, the value of the objective function is in a stable state after about 260 calculations. Within 100 iterations, the algorithm converges rapidly and finds the extreme value in the local region from 100 to 250 times. It can be seen from Table 2 that the average error obtained by the four methods is all less than 0.1 pixel, which is due to the high accuracy of the experimental instrument in this paper. But relatively speaking, the calibration error value of this paper is 0.026 pixel, which is better than the 0.105 pixel value of Zhang's calibration method and less than the 0.075 pixel value of grey wolf algorithm and the 0.058 pixel value of particle swarm optimization algorithm.

## Conclusion and future works

Based on the idea of bionics, this paper establishes a gray wolf optimization algorithm model based on Levy flight and mutation mechanism. The algorithm is applied to the field of 3D reconstruction engineering. The nonlinear solution problem in the camera calibration process is solved. Through field experiments and data processing, the following conclusions are drawn:

1. Using the principle of bionics, the classical calibration algorithm is combined with the gray wolf algorithm. It effectively avoids the problem that the traditional calibration algorithm is easy to fall into the local optimal solution. The stability and robustness of the camera calibration algorithm are greatly improved.
2. Add Levy flight and mutation mechanism to the gray wolf optimization algorithm model. Optimize the convergence and population diversity of the algorithm. This can make the algorithm suitable for the actual camera calibration calculation, and effectively improve the calibration accuracy and stability.
3. Through the optimization algorithm model established in this paper, the parameter matrix of the camera is solved nonlinearly. The average error of reprojection of the calculation results is 0.026 pixels, which is better than that of PSO, GWO and other methods.

In the follow-up work, the research group will also conduct a detailed analysis of the reconstructed calibration point errors. The influence of the optimization algorithm model parameters on the calibration results is studied. The algorithm is then optimized and improved for a better accuracy and convergence speed of the camera calibration algorithm. In addition, the method in this paper has only been verified in the camera calibration calculation for the time being. In the future, we will continue to explore the feasibility of applying the algorithm in this paper to other fields.

It is worth mentioning that the work in this paper is helpful for the application of bionic optimization algorithms in the field of camera calibration, such as DMO, EOS, RSA, etc. mentioned above. This will provide a reference for future scholars' work to apply these excellent bionic algorithms to camera calibration.
